# Change in economy of ultrasound probe motion among general medicine trainees

**DOI:** 10.1186/s13089-023-00345-2

**Published:** 2024-01-30

**Authors:** Gerard Salame, Matthew Holden, Brian P. Lucas, Albert Portillo

**Affiliations:** 1 Department of Medicine, Saint Joseph Hospital/SCL Health, 1375 E 19th Ave, Denver, CO 80218 USA; 2 School of Computer Science, Carleton University, Ottawa, ON Canada; 3 Medicine Service, White River Junction VA Medical Center, White River Junction, Vermont, USA; 4 The Dartmouth Institute of Health Policy and Clinical Practice, Geisel School of Medicine at Dartmouth College, Hanover, NH USA; 5 Geisel School of Medicine at Dartmouth College, Hanover, NH USA; 6 AP Engineering Research and Development, Denver, USA

**Keywords:** Motion economy, Point of care ultrasound, Image acquisition, Cardiac ultrasound, Point of care ultrasound training/proficiency

## Abstract

**Objectives:**

To observe change in economy of 9 ultrasound probe movement metrics among internal medicine trainees during a 5-day training course in cardiac point of care ultrasound (POCUS).

**Methods:**

We used a novel probe tracking device to record nine features of ultrasound probe movement, while trainees and experts optimized ultrasound clips on the same volunteer patients. These features included translational movements, gyroscopic movements (titling, rocking, and rotation), smoothness, total path length, and scanning time. We determined the adjusted difference between each trainee’s movements and the mean value of the experts’ movements for each patient. We then used a mixed effects model to trend average the adjusted differences between trainees and experts throughout the 5 days of the course.

**Results:**

Fifteen trainees were enrolled. Three echocardiographer technicians and the course director served as experts. Across 16 unique patients, 294 ultrasound clips were acquired. For all 9 movements, the adjusted difference between trainees and experts narrowed day-to-day (*p* value < 0.05), suggesting ongoing improvement during training. By the last day of the course, there were no statistically significant differences between trainees and experts in translational movement, gyroscopic movement, smoothness, or total path length; yet on average trainees took 28 s (95% CI [14.7–40.3] seconds) more to acquire a clip.

**Conclusions:**

We detected improved ultrasound probe motion economy among internal medicine trainees during a 5-day training course in cardiac POCUS using an inexpensive probe tracking device. Objectively quantifying probe motion economy may help assess a trainee’s level of proficiency in this skill and individualize their POCUS training.

**Supplementary Information:**

The online version contains supplementary material available at 10.1186/s13089-023-00345-2.

## Introduction

Evaluating point of care ultrasound (POCUS) trainee skill in image acquisition often requires direct supervision. Yet, this approach is subjective, prohibitively time consuming at most institutions [1], and focuses more on the quality of the final image than the motor skills that are involved in obtaining the image [2].

Recently, Ackil et al. [3] used hand motion analysis (HMA) to monitor skill proficiency and decay among paramedics undergoing training in cardiopulmonary POCUS. HMA examines how efficiently a task is accomplished, otherwise known as motion economy [4]. HMA has been used successfully to assess proficiency in the development of surgical skills among trainees [5]. For example, the Imperial College Surgical Assistant Device (ICSAD) is a commercially available motion tracking tool that has been validated to assess motion economy among surgical and anesthesia trainees [6, 7], as well as create competency-based learning curves [8]. Despite the uptake of motion economy studies to track the development and competency of surgical trainees, probe movement analysis has seldomly been used to evaluate image acquisition in POCUS training.

We built a novel inexpensive device to track probe motion called Probe Watch™ which is able to record multiple features of ultrasound probe motion in real time. In this study, we used Probe Watch™ to explore how the differences in probe motion economy between trainees and experts narrowed over a 5-day POCUS training course.

## Materials and methods

### Overview

We conducted a prospective study of internal medicine trainees enrolled in a 5-day elective in cardiopulmonary POCUS at a single hospital in the academic years 2020–21. Our goal was to track differences in ultrasound probe movements between trainees and experts during the training course. We recorded nine features of probe movement with the Probe Watch™ during image acquisition of three different cardiac ultrasound views by both trainees and experts on the same patients within minutes of each other. We tracked differences between trainees and experts over the course of the elective. Because no identifying information was recorded for trainees or patients, the SCL Health Review Board required only verbal consents.

### Setting and participants

The Saint Joseph Internal Medicine (IM) Training Program in Denver, Colorado, USA, has 38 trainees in post graduate years (PGY) 1 through 3. The 5-day POCUS course includes supervised and unsupervised scanning of volunteer patients, as well as 2 h-long lectures dedicated to image acquisition in cardiac POCUS. All trainees enrolled in the course during the study period (4 groups of 2–5 trainees) agreed to participate in the study. Trainees could only take the course once during their residency. The expert cohort included the course instructor, who had 12 years of POCUS teaching experience, and 3 volunteer echocardiography technicians from the Department of Cardiology. The instructor selected and verbally consented a single patient each day from a convenience sample of patients on the general medicine and surgery wards. All ultrasound clips obtained on a given day (by both trainees and experts) were acquired from the same patient. Each patient was enrolled only once during the study.

### Acquisition protocol

**Fig. 1:**
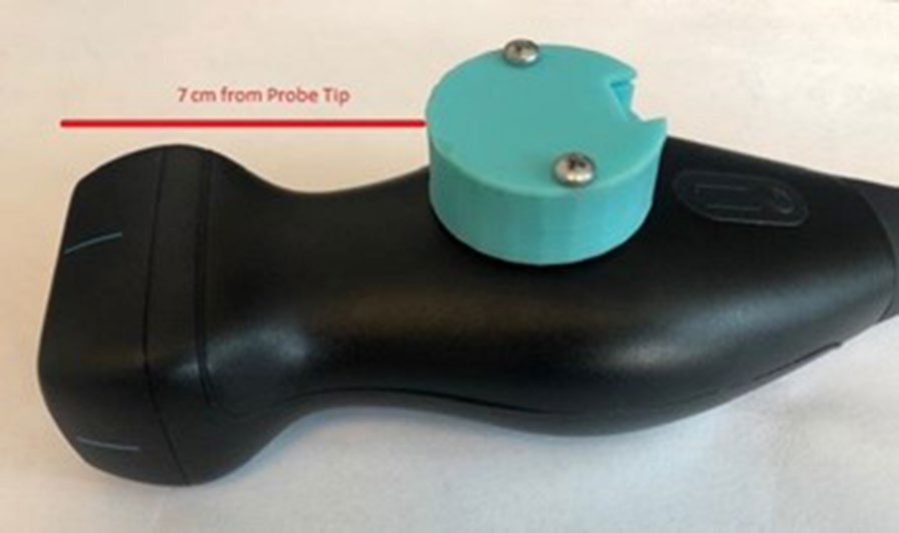
Probe with tracker

**Fig. 2:**
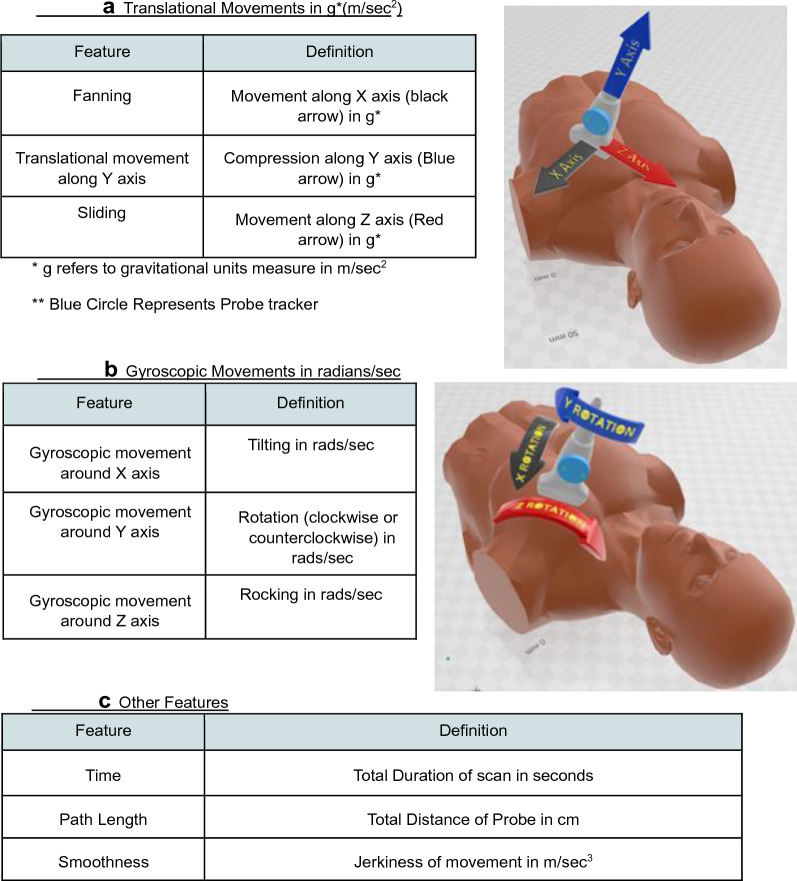
Definition of probe features

Patients were placed in the left lateral decubitus position and asked to maintain this position throughout acquisitions by all operators. Operators were not allowed to reposition the patient but were free to optimize the ultrasound image as they saw fit. Trainees and experts used the same handheld Butterfly IQ™ ultrasound device, which was set to deep cardiac mode (1 to 5 MHz) and equipped with a Probe Watch™ device that was wired to a computer (Fig. 1). Details about the Probe Watch™ are provided in Holden et al. [9]. In brief, the Probe Watch™ is a device that consists of a magnetometer, an accelerometer, and a gyroscope. It maps three-dimensional (3D) movements of the probe in real time by recording 9 movement features (Fig. 2) [10]. To minimize the impact of the of the Probe Watch™ on image acquisition it was placed 7 cm from the end of the ultrasound probe. The Probe Watch itself weighs 1.2 oz (compared to the probe which weighs 11 oz).

The three-dimensional planes of movement were defined relative to the center of the ultrasound probe. Translational and gyroscopic velocities were measured for each axis Translational acceleration along the *x*, *y* and *z* planes were defined as fanning, compression and sliding, respectively, and measured in gravitational units (*g* in cm/s^2^). The total translational path length of the probe was calculated using this data. Gyroscopic movements along the *x*, *y* and *z* planes represented: tilting, rotation, and rocking, respectively, and were measured in radians (rads/s). The time to completion of image acquisition, was recorded in seconds. The cumulative translational acceleration and rotational velocities were measured and used to calculate the smoothness of movement (in m/s^3^). Smoothness of movement describes the rate at which acceleration changes with respect to time: higher values suggest high-amplitude (or “jerky”) probe movements while lower values reflect low-amplitude (or intentional) probe motion. Movement data were saved in real time on the investigator’s computer that was connected to the Probe Watch™ via USB cable, and later processed using the open-source program 3D Slicer™. The processing generated a single numerical value for each of 9 movement features from each clip acquisition.

Each operator obtained 3–6 s ultrasound clips of the parasternal long axis (PLAX), parasternal short axis (SAX), and 4 chamber apical (AP) views of the heart. Experts acquired clips first, while trainees waited outside patient rooms. Trainees individually and sequentially entered the room and obtained clips without any feedback from the course director (who is also the course instructor), who remained in the room for data recording. Trainees were asked to scan one patient per day. To ensure that the director did not interfere with the trainees’ performance, the director had his back turned to both trainee and patient and did not offer any guidance. For each clip, after the ultrasound probe was placed on the chest, trainees signaled when to begin Probe Watch™ recordings; and ended them after obtaining the best 3 to 6 s ultrasound clip possible. Clips were automatically de-identified by the Butterfly IQ™ software and stored in the Butterfly IQ™ encrypted cloud server. Ultrasound gel was removed from the patient’s chest in between ultrasound operators.

Blinded to operator and patients, the quality of each clip was later assessed by the course director using an image quality assessment instrument (Additional file 1: Fig. S1). This instrument was specifically created for this training course and was designed to assess if clips contained the correct structures in the correct axis and orientation. Each movement feature from each acquired clip was assigned a single numerical value. When more than one expert acquired clips on the same patient, a mean value was generated. We determined the differences between trainees and experts by subtracting the expert values from the trainee values. Because higher values of each movement feature reflected more movement, positive values in differences indicate lower trainee performance. In contrast, values near zero indicate that trainees’ movements were similar to the experts (Table 2).

### Statistical analysis

We created ten separate two-level random-intercept linear mixed models—one for each movement feature and one for image quality. The dependent variable (*Y*-variable) in each model was the adjusted difference between trainees and experts. The effect of time (day of training) was assumed linear, and the correlations between repeated measurements within-subject were described with an unstructured variance–covariance matrix. Additional variables included in the model were imaging window (para-sternal long axis, para-sternal short axis, or four-chamber apical) and trainee postgraduate level. We used the Holhm–Bonferroni method to define statistically significant *p* values that would account for multiplicity as well as their corresponding confidence intervals [11] (A complete list is available in Additional file 1: Table S1).

In developing this statistical model, we assumed the following. First, all participants were blinded to each other’s performance, ensuring that the image acquisition process between ultrasound operators was independent. Second, since the same patient was scanned by both expert and trainee groups minutes apart and because patient repositioning was prohibited, the difference in performance between and within ultrasound operators was ascribed to the variable skill levels in image acquisition. Third, the expert value for each movement feature represented the best possible performance for that metric.

## Results

A total of 15 trainees (6 PGY-1 s, 6 PGY-2 s, and 3 PGY-3 s) acquired 53 sets of ultrasound clips from 16 unique patients. Each trainee scanned 3–4 patients (mean 3.5), acquiring 10–11 ultrasound clips (mean 10.5) per elective rotation for a total of 159 clips (Tables 1, 2). Experts acquired a total of 198 clips, all 16 patients were scanned by the POCUS director among them 7 had clips acquired by an echocardiographer. Because each clip was associated with 10 features (9 from the probe tracker and one form the image score), a total of 3570 data points were analyzed.

**Table 1:** Description of trainee and expert cohorts

Group	Participants per group	Total number of patients scanned	Total number of acquired clips	Total number of data points
*Trainees*				
PGY 1	6	21	10.5 (63)	630
PGY2	6	22	11 (66)	660
PGY 3	3	10	10 (30)	300
Total	15	53	10.5 (159)	1590
*Experts*				
Echo Tech	3	21	21 (63)	630
POCUS director	1	16*	135*	1350
Total	4	37	49.5 (198)	1980
*Overall*				
Total	19	119	294	3570

*PGY* post graduate level

Trainee cohort: All residents participating in the POCUS elective

Expert cohort: Combination of POCUS director + 3 echocardiography technicians (echo tech)

*Given *n* = 1, the mean is the same as the total in this group

**Table 2:** Summary of the change in feature difference between expert and trainees cohort

Metric	Change in the difference of feature (trainee minus expert) per day*	Confidence intervals accounting for multiplicity**
*Gyroscopic movements in rads/s*		
Along *X* axis or tilting	− 10.9 rads/s/day	− 19.31–4.21 rads/s/day
Along *Y* axis or rotation	− 9.14 rads/s/day	− 19.00 to − 4.43 rads/s/day
Along *Z* axis or rocking	− 4.66 rads/s/day	− 9.53 to − 0.02 rads/s/day
*Translational movements (in cm/s*^2^)		
*X* path or fanning	− 7.53 cm/s^2^/day	− 15.42 to 0.33 cm/s^2^/day
*Y* path or compression	− 1.52 cm/s^2^/day	− 2.67 to − 0.374 cm/s^2^/day
*Z* path or sliding	− 1.64 cm/sec^2^/day	− 2.90 to − 0.84 cm/s^2^/day
*Other probe motion data*		
Smoothness (m/s^3^)	− 0.47 (m/s^3^) /day	− 0.85 to − 0.11 m/s^3^ /day
Study time (s)	− 6.32 s/day	− 10.98 to − 1.66 s/day
Total path (cm)	− 3.12 cm/day	− 6.2 to − 0.50 cm/day

*Change in feature per day represents the change in the adjusted difference of performance metrics between the trainees and expert cohort for each ultrasound elective day. Negative change means that the gap between trainees and experts narrowed

**Confidence intervals were calculated using statistical significance of *p* values based on the Holhm–Bonferroni correction for multiplicity

***Did not reach statistical significance

**Fig. 3:**
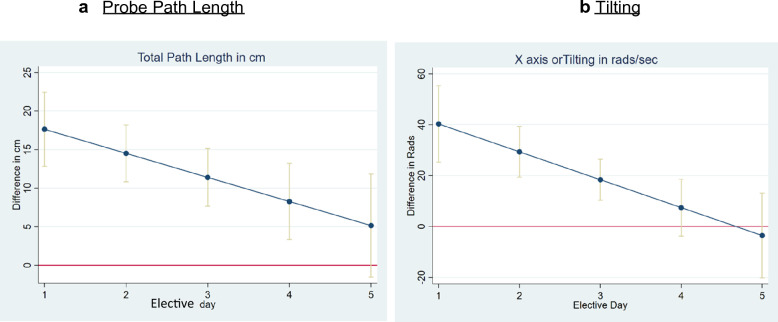
Change in the difference between expert mean and trainees cohort probe movements. When confidence intervals include 0 (indicated by the red line) there is no longer any statistically significant difference in the performance metric between the trainees and expert cohorts. **a** Total probe path length in cm, coefficient = − 3.12 cm, *p* = 0.006, CI [(− 5.35)–( − 0.89)] **b** Tilting in rads, coefficient = − 10.96 rads/day, *p* = 0.02 CI: [(− 19.3)–( − 4.21)]

Table 2 shows the average daily change in adjusted difference between trainees and experts for all nine movement features, as well as image score. For all nine movement features, the daily improvement was statistically significant. For example, the adjusted difference between trainees and experts in path length changed by − 3.12 cm (95% CI −6.2 to −0.50 cm) per day. This means that, on average, the total path length difference between trainees and experts narrowed by 3.12 cm in total probe path length for each day residents were on the elective (Fig. 3a) By elective day 5, adjusted differences between trainee and expert probe movements were no longer statistically significant. (Additional file 1: Fig. S2).

With regard to the image score, we could not find any statistically significant linear trend in the average difference between experts and trainees across all timepoints (− 1.5 s/day, 95% CI [− 5.33, 2.3]).

## Discussion

Higher level proficiency is associated with the ability to optimize the image using the fewest number of probe movements HMA is more reliable than subjective expert assessments [12] and correlates with objective structured evaluation of technical skills [13]. It is utilized by the Imperial College System Assessment Device (ICSAD) to assess proficiency across multiple procedural tasks and specialties [5, 6, 14–17]. Currently, ascertaining proficiency in POCUS involves the subjective assessment by a POCUS instructor present at the time of trainee image acquisition. This approach creates interactions between the rater, trainee and patient that confound the raters’ final assessment [18]. These interactions are significantly reduced by applying objective assessments method (such as a checklist) [19]. Although these objective methodologies are reproducible (such as the Ultrasound Competency Assessment Tool [2] or Rapid Assessment of Competency in Echocardiography [20]) they focus mainly on image quality and thus fail to consider the intricate probe motions needed to acquire an ultrasound image, especially when patient anatomy or body habitus impact image acquisition. Ideally, instructors should directly observe their trainees during image acquisition. However, the scarcity of qualified POCUS educators limits the implementation of this approach [21]. There is evidence that contemporaneous POCUS scanning by trainer and trainees can be used for quantitative assessment of image acquisition skill [22]. This approach optimizes the instructor’s time by requiring them to obtain the image only once.

In this study, we combined the use of objective probe tracking data and contemporaneous scanning. To our knowledge, this is the first study that assessed the serial change in ultrasound probe motion between experts and residents during a POCUS elective. In a prior publication, we demonstrated that probe motion analysis during POCUS imaging offers insight into a trainee’s skill level [9]. In this study, we trended the adjusted difference in 10 performance metrics between internal medicine residents and expert POCUS operators during a 5-day POCUS elective. (Fig. 3) We demonstrated that all nine probe movements showed a statistically significant daily decrease in this difference (Table 2) without any detriment to image quality. Currently, the assessment of POCUS competency relies heavily on number of images acquired. This threshold varies significantly between and within specialties, is based on expert opinion and does not consider the individual differences in the pace at which trainees learn a specific skill [23].

Except for scanning time, the difference in probe motion between the expert and trainee cohorts was no longer statistically significant by day 5 of the elective, suggesting that trainee’s probe motion was approaching that of the expert cohort. (Fig. 3, Additional file 1: Fig. S2) This reflects the impact of repetitive instruction and practice in image acquisition during the POCUS elective. By elective day 5, trainees would have received a total of 32 h of instruction and practice in bedside ultrasound. A study by Lucas et al. [24] demonstrated that after 27 h of practice in bedside cardiac ultrasound, hospitalists demonstrated excellent diagnostic accuracy for the identification of common cardiac abnormalities. This time frame of ultrasound training may signal a threshold at which trainees successfully incorporate instructions that lead to significant changes in their probe movement behavior, e.g., consistent anchoring of their hand on the patient, intentional movements for image optimization. In the future this information can be used to compare different teaching strategies.

### Strength and limitations

Our study has several strengths. The random selection of volunteer patients prior to assessing their cardiac windows minimizes selection bias and parallels real life experience. Contemporaneous image acquisition by expert and trainee operators on the same patient, blinding the participants to each other's performance and patient immobility between ultrasound operators ensured that day to day changes in performance metrics in the trainees’ group was attributed to the impact of POCUS training on their image acquisition skills.

Our study had several limitations. First, data were collected in only 4 out of the 5 days of the elective as each resident had one day of outpatient internal medicine clinic. This led to differences in the number of clips obtained for each elective day which was most pronounced on elective day 5. Second, although the course director obtained clips for all the studies, 8 of the 15 patients were not scanned by the echocardiography technicians. On those occasions the expert mean performance was reduced to the course director’s metrics. Third, the image score used was created with the aid of 8 national POCUS experts but has not been validated. Fourth, no follow-up was performed to evaluate skill decay and retention. Fifth, because of resident scheduling issues we were only able to enroll 3 third year residents. Although patients were asked to remain still it is conceivable that small drifts in patient positioning occurred during transition between ultrasound operators and affected image acquisition. Finally, all features showed a large within operator residual variance, suggesting that other variables such as prior ultrasound experience, patient body habitus and comorbidities may need to be included in future studies.

## Conclusion

Using an inexpensive device, we successfully recorded the change in ultrasound probe motion during image acquisition of 3 common cardiac views over a 5-day POCUS elective among internal medicine resident trainees. Using objective parameters for probe motion assessment and contemporaneous image acquisition by both the expert and trainee cohorts avoids the potential for unintended biases, optimizes POCUS instructor time, and accounts for the variability in patient anatomy. This makes it possible to identify patterns in image acquisition among trainees and tailor their POCUS training, all the while decreasing direct supervision by POCUS instructors. Future studies will assess whether economy of movement data can be used to create expected learning curves to tailor training, to define objective thresholds for competency evaluations, and to assess the impact of educational interventions.

### Supplementary Information

**Additional file 1: Fig. S1.** Image score. **Fig. S2.** Change in the difference between expert mean and trainees cohort Probe movements. **Table S1.** *P* values cutoff for statistical significance accounting for multiplicity using the Holhm–Bonferroni method.

## Data Availability

Raw data are made available to editors upon request.

## Abbreviations

POCUS: Point of care ultrasound
HMA: Hand motion analysis
ICSAD: Imperial College Surgical Assistant Device
IM: Internal medicine
PGY: Post graduate year
PLAX: Parasternal long axis
SAX: Parasternal short axis
AP: Apical 4 chamber
