# Supplementary material for: Change in economy of ultrasound probe motion among general medicine trainees
Source: Ultrasound J. 2024 Jan 30;16:5. doi: 10.1186/s13089-023-00345-2 (PMC10828286; doi:10.1186/s13089-023-00345-2)
Supplement: Supplementary file 1 — Additional file 1: Fig. S1. Image score. Fig. S2. Change in the difference between expert mean and trainees cohort Probe movements. Table S1. P values cutoff for statistical significance accounting for multiplicity using the Holhm–Bonferroni method. [file 13089_2023_345_MOESM1_ESM.docx]

**Additional file**

**Figure S1: Image Score**

A scoring table was developed for each of the 3 cardiac views: Parasternal Long Parasternal Short Axis and Apical windows. An image score was retrospectively calculated by the primary investigator. View specific structures in their proper axis and orientation were awarded 10 points and their absence was given 0 points (light blue cells). Images were penalized 10 points if common view specific errors were noted (red cells). The total score was tabulated as a final percentage

**Image score tables**

|  | | | \| Parasternal Short Axis \| \| \| --- \| --- \| \| Structure \| Points \| \| Gain \| 10 \| \| Depth \| 10 \| \| RV (+/-Free Wall) \| 0 \| \| IVS \| 0 \| \| LV Free Wall \| 0 \| \| LV inf Wall \| 10 \| \| Posterior pericardium \| 10 \| \| Elliptical LV \| 0/ (-10) \| \| Total \| 40 \| \| **Image Score** \| **57%** \| | \| 4 Chamber Apical View \| \| \| --- \| --- \| \| Structure \| Points \| \| Gain \| 10 \| \| Depth \| 10 \| \| LV \| 10 \| \| LA \| 10 \| \| RV \| 10 \| \| RA \| 10 \| \| MV \| 10 \| \| TV \| 10 \| \| LV Apex \| 10 \| \| Foreshortened LV \| 0/ (-10) \| \| Oblique IVS \| 0/ (-10) \| \| Total \| 90 \| \| **Image Score** \| **100%** \| |
| --- | --- | --- | --- | --- | --- | --- | --- | --- | --- | --- | --- | --- | --- | --- | --- | --- | --- | --- | --- | --- | --- | --- | --- | --- | --- | --- | --- | --- | --- | --- | --- | --- | --- | --- | --- | --- | --- | --- | --- | --- | --- | --- | --- | --- | --- | --- | --- | --- | --- | --- | --- | --- | --- | --- | --- | --- | --- | --- |
|  | Parasternal Long Axis | |  |  |
|  | Structure | Points |  |  |
|  | **Gain** | **10/0** |  |  |
|  | Depth | **10/0** |  |  |
|  | RVOT | **10/0** |  |  |
|  | IVS | **10/0** |  |  |
|  | LVOT | **10/0** |  |  |
|  | AV | **10/0** |  |  |
|  | LA | **10/0** |  |  |
|  | MV | **10/0** |  |  |
|  | LV | **10/0** |  |  |
|  | Post Pericardium | **10/0** |  |  |
|  | Desc Ao | **10/0** |  |  |
|  | LV foreshortened | 0/ (-10) |  |  |
|  | TV seen in |  |  |  |
|  | RVOT | 0/ (-10) |  |  |
|  | Oblique IVS | 0/ (-10) |  |  |
|  | Total | 100 |  |  |
|  |  |  |  |  |

Legend: RVOT: Right Ventricular Outflow Tract. LVOT: Left Ventricular Outflow Tract AV: Aortic Valve; MV: Mitral Valve; TV: Tricuspid Valve; RV: Right Ventricle; LV : Left Ventricle; IVS: Interventricular

Septum; LA: Left Atrium; RA: Right Atrium; Desc Ao: Descending Aorta

Figure S2: Change in the difference between expert mean and trainees cohort Probe movements

**1. Gyroscopic Movements**


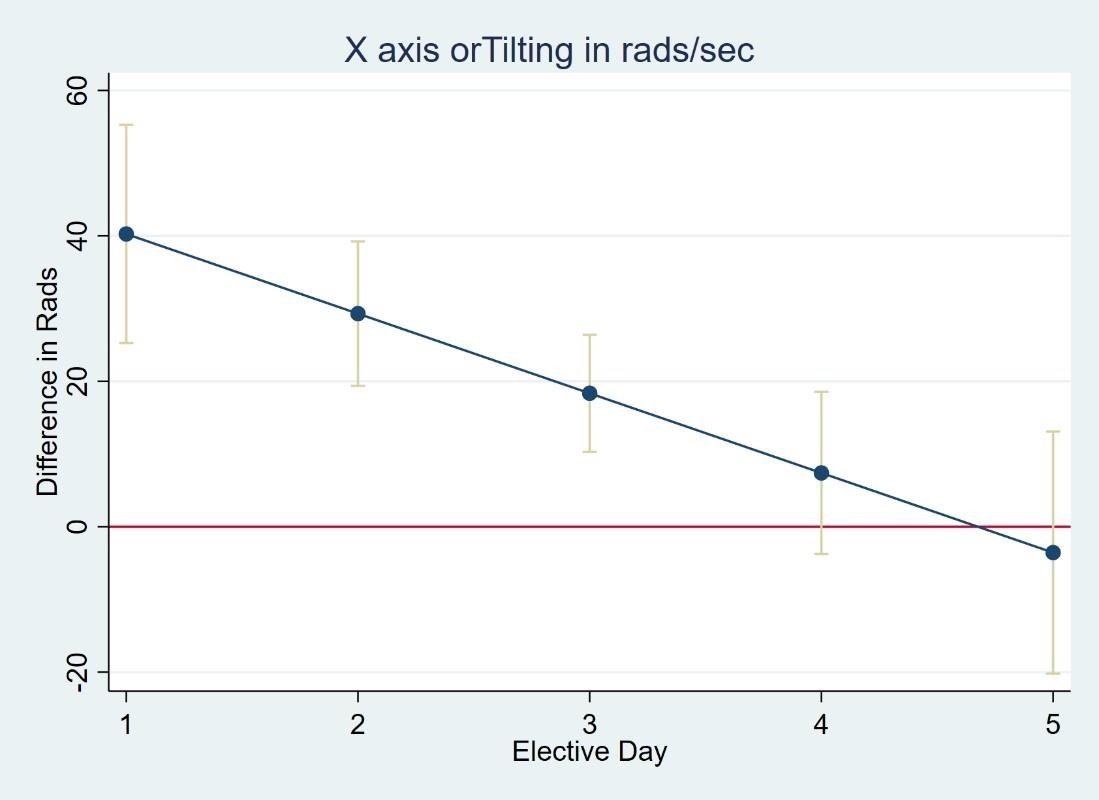

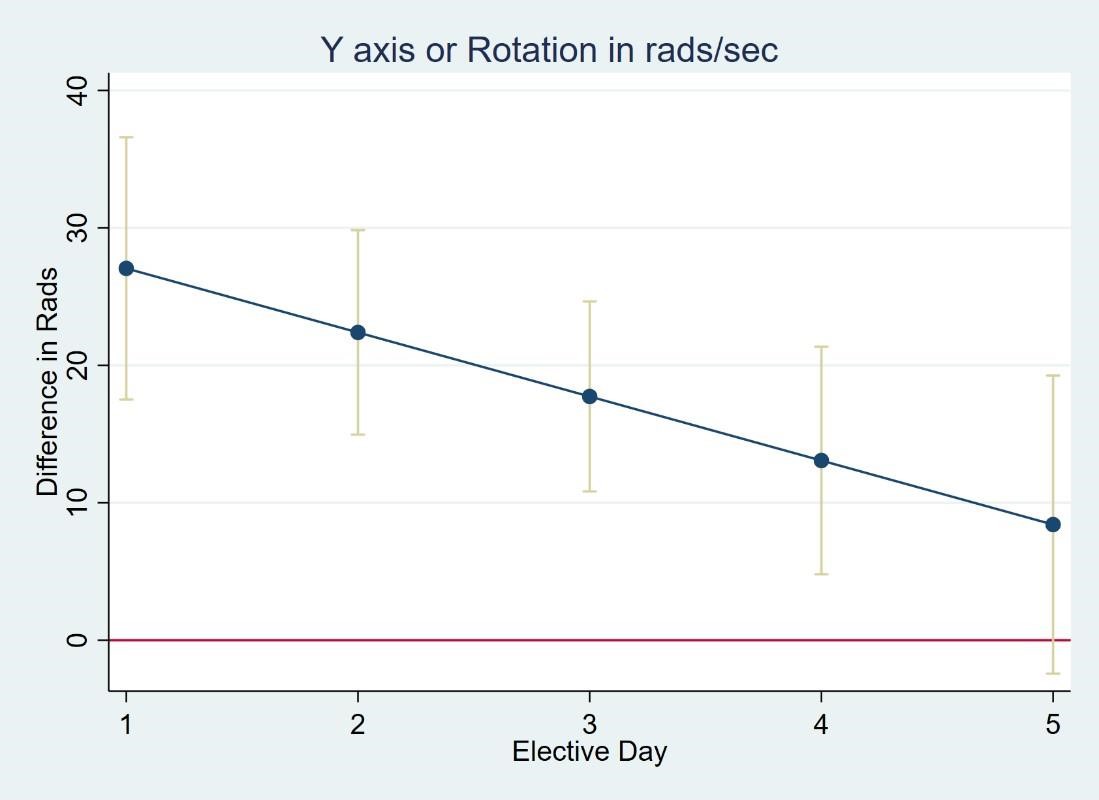


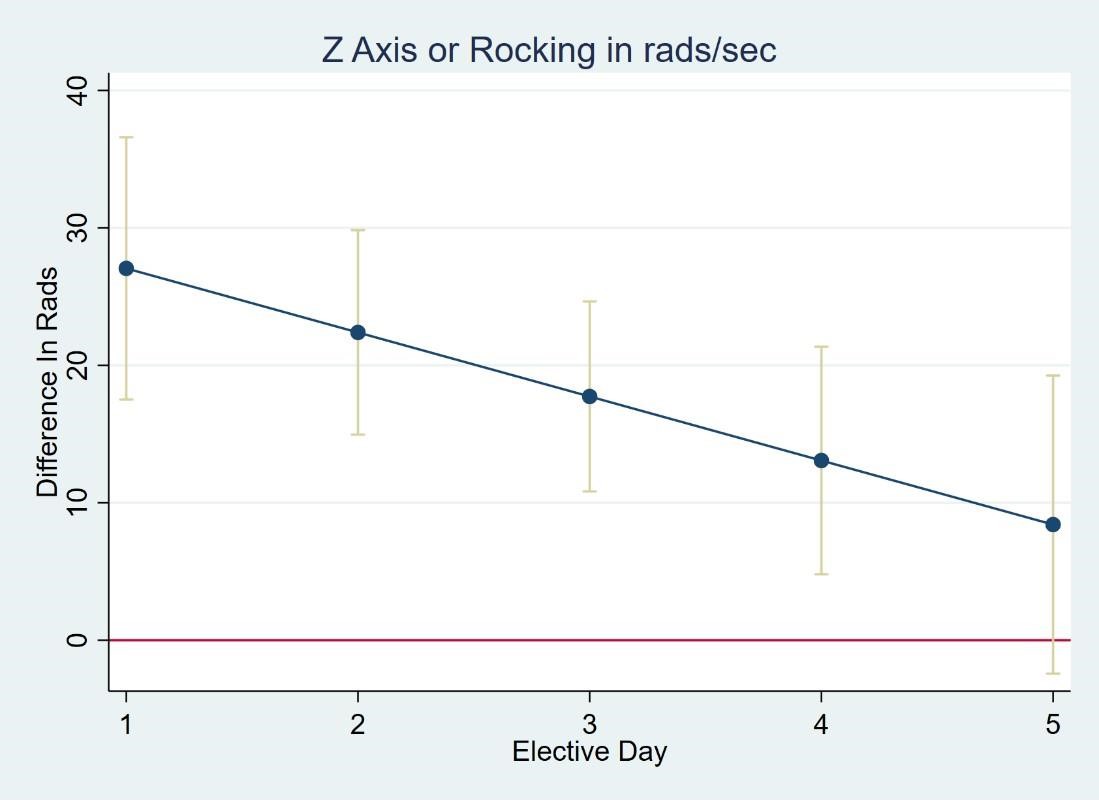


**2.Translational Movements:**


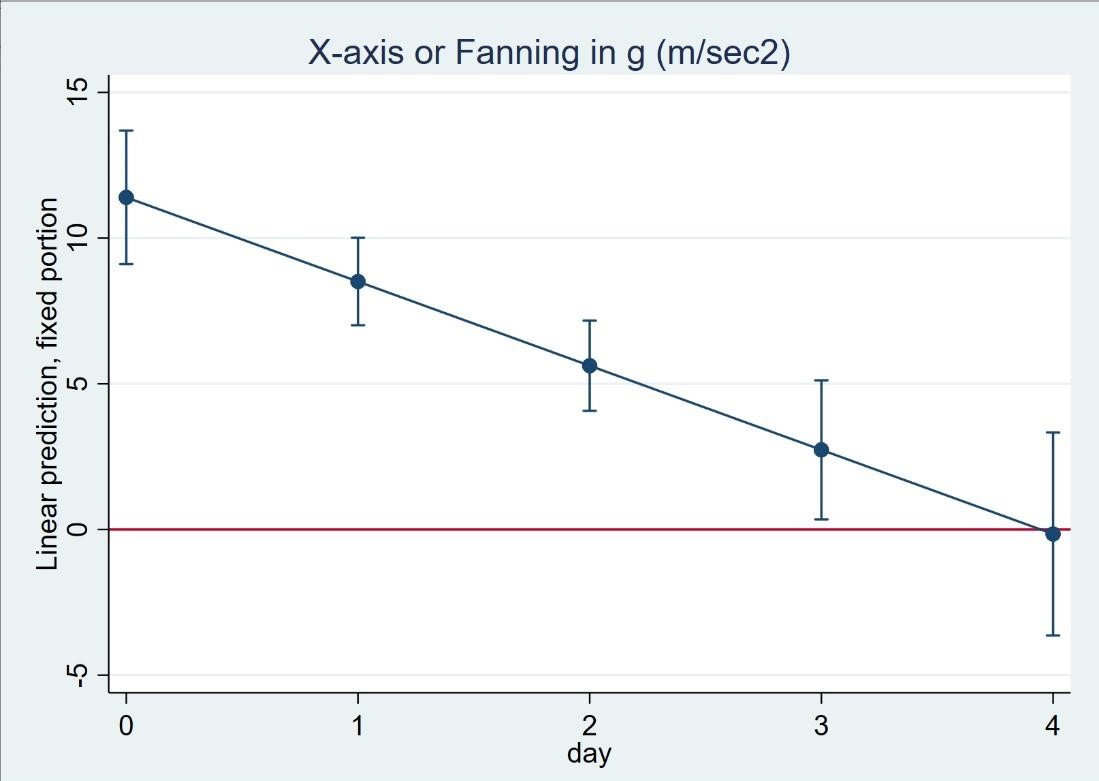


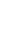

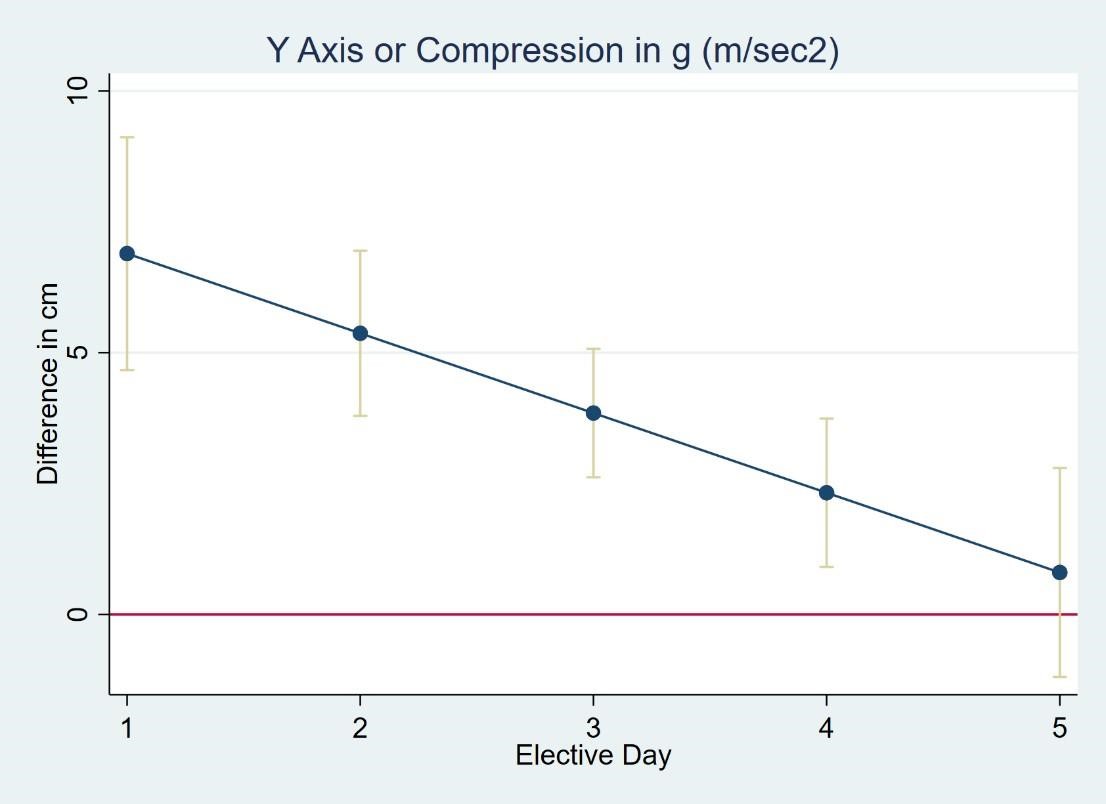

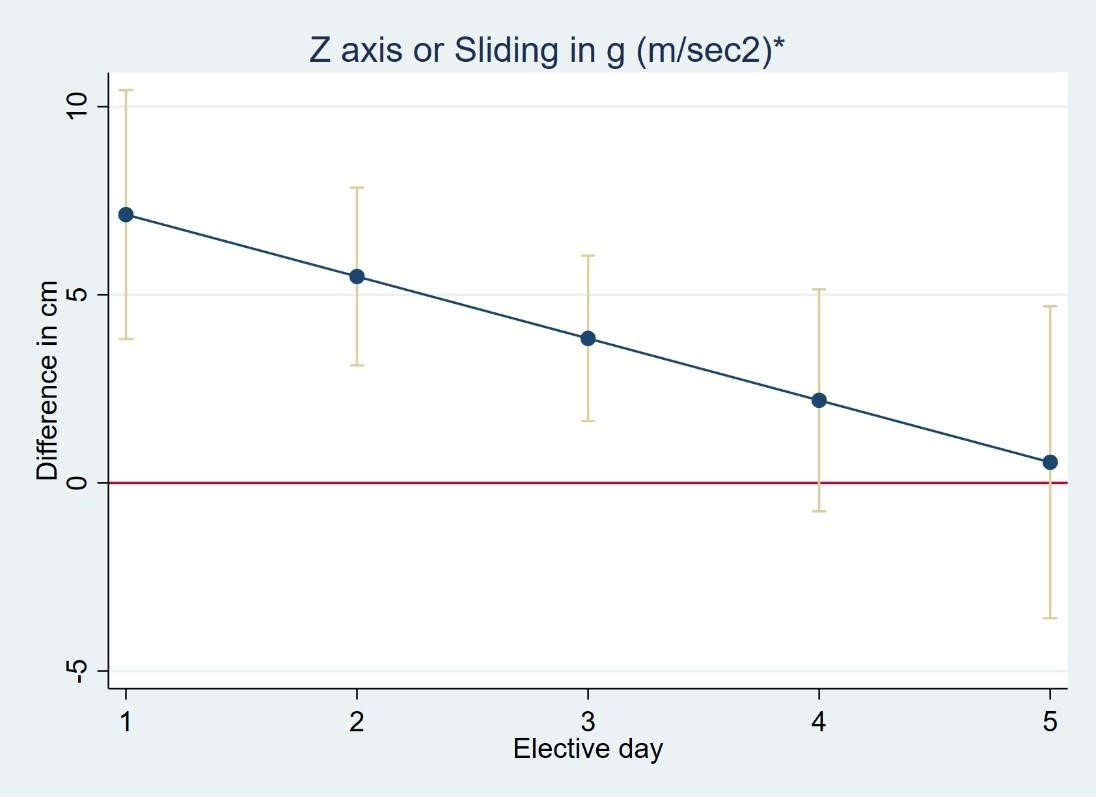


**3. Miscellaneous:**


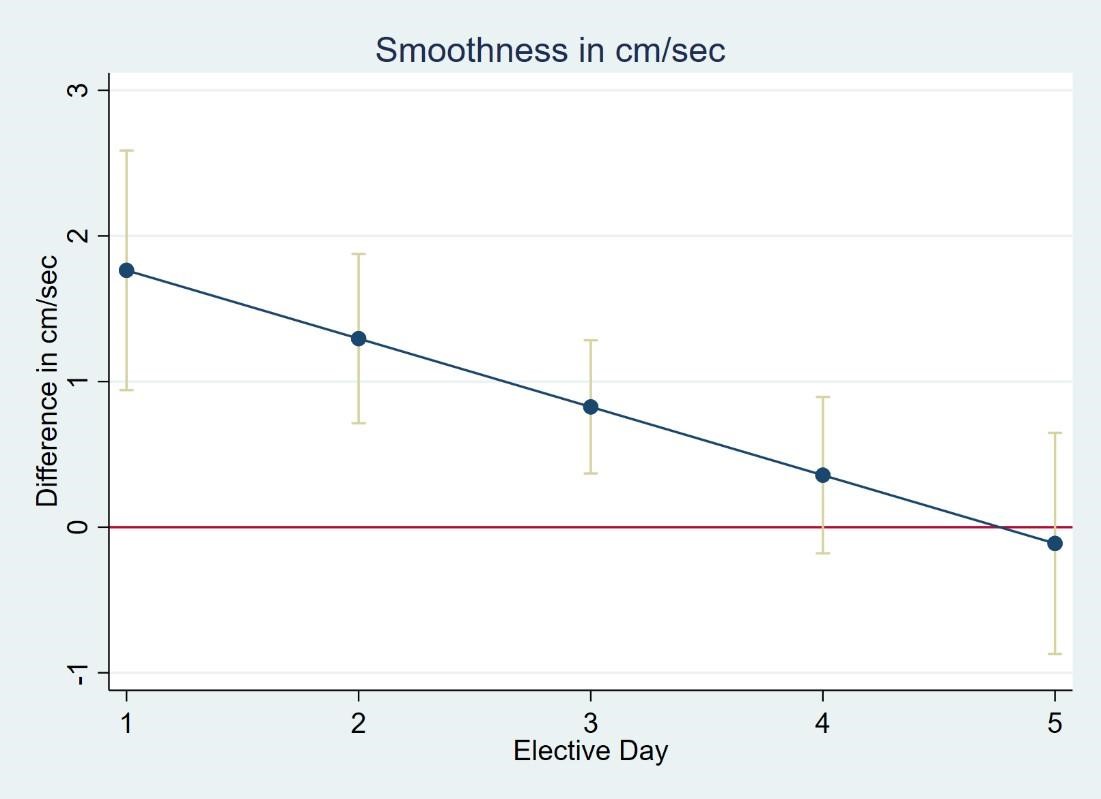


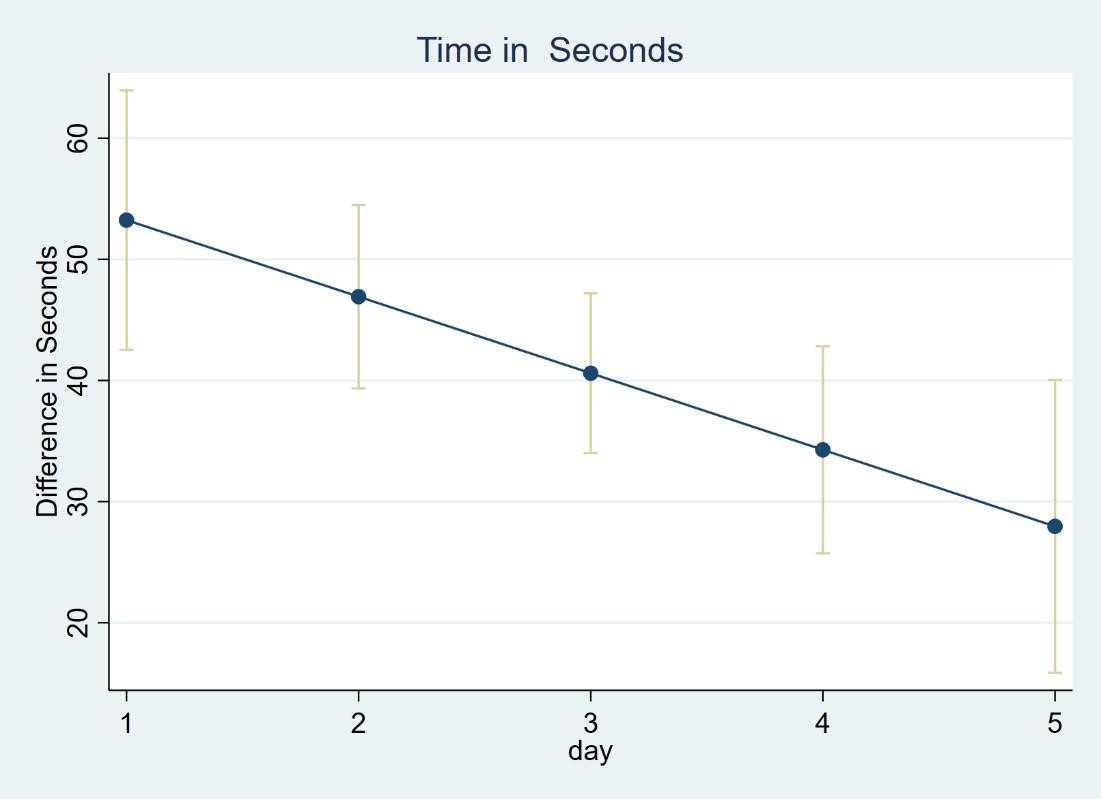


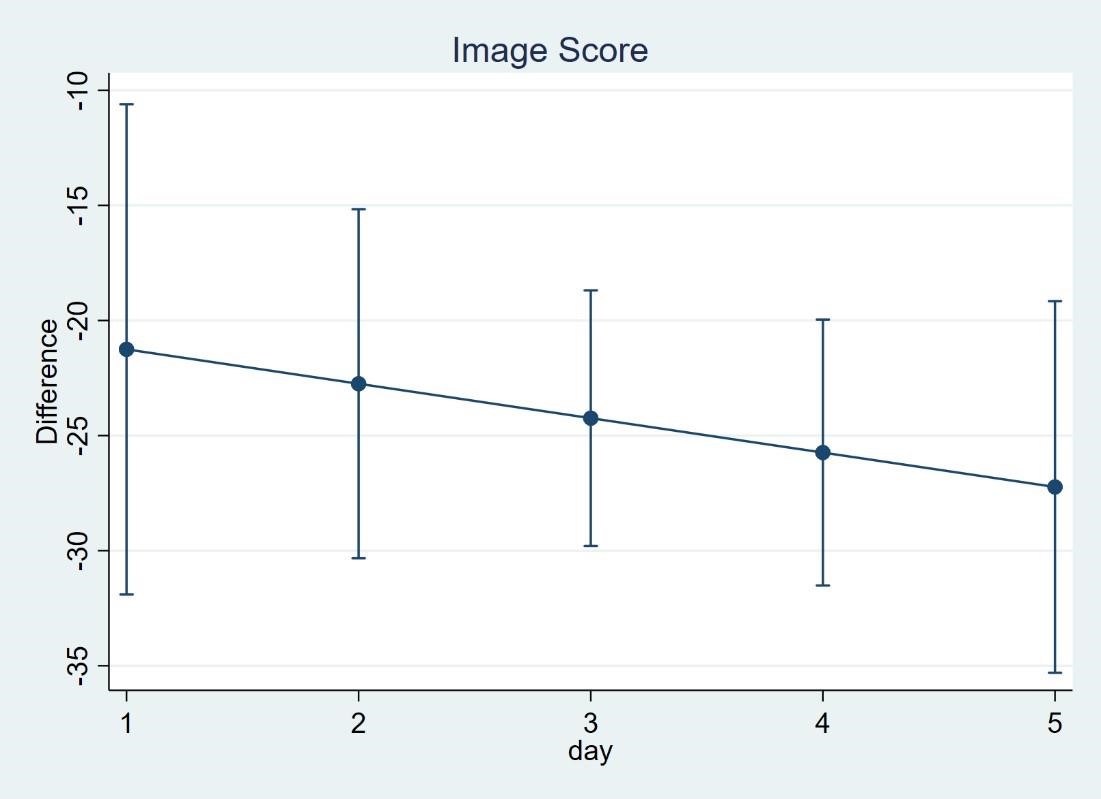

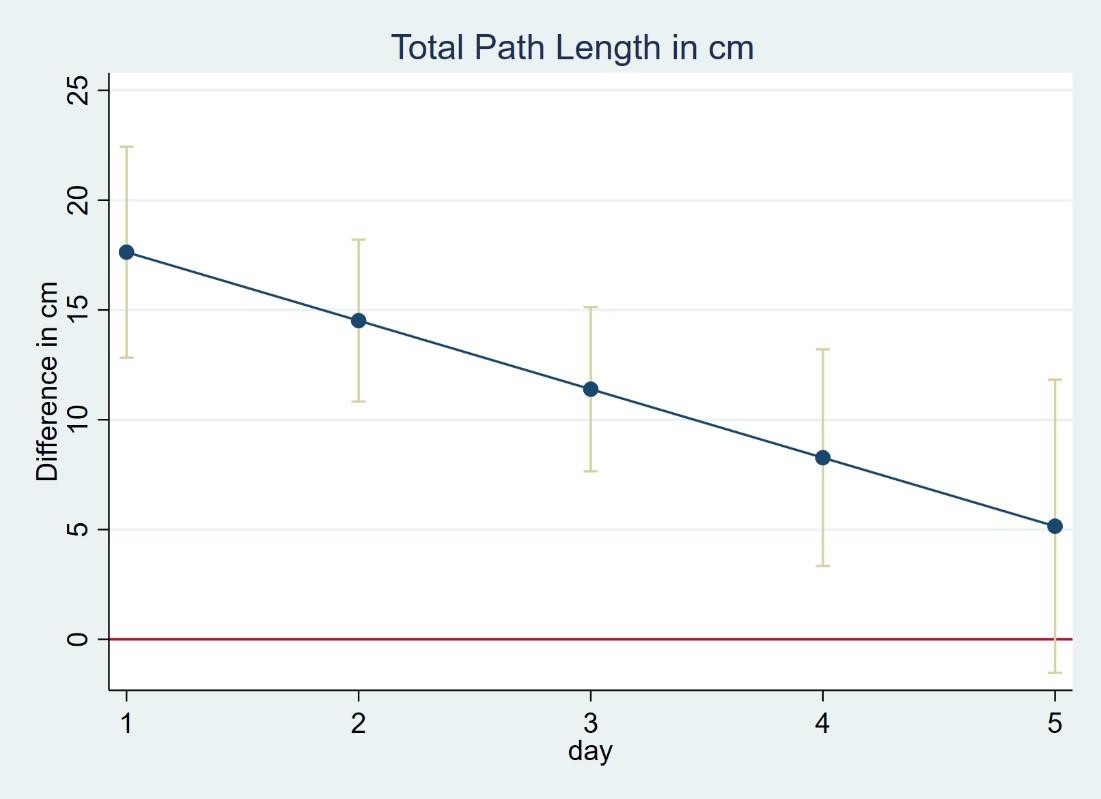


Red Reference lines: When present and included inside the CI brackets indicate the day when the difference in performance metrics between the expert and novice cohorts was no longer statistically significant.

All differences here refer to the adjusted or model difference not raw difference

Table S1: Table of p-values cutoff for statistical significance accounting for multiplicity using the Holhm-Bonferroni method^1^

| Feature | p-value | Definition of statistical significance of p-values accounting for multiplicity * | Confidence Intervals adjusted for multiplicity* |
| --- | --- | --- | --- |
| Rotation | 0 | 0.006 | -19.00 to -4.43 rads/sec/day |
| Tilting | 0 | 0.006 | -19.31-4.21 rads/sec/day |
| Y Path Length | 0 | 0.007 | -2.67 to -.374 cm/sec^2^/day |
| Smoothness | 0.001 | 0.008 | -.85 to -.11 m/sec^3^ /day |
| X Path Length | 0.002 | 0.01 | -15.42 to .33 cm/sec^2^/day |
| Z Path Length | 0.005 | 0.012 | -2.90 to -.84 cm/sec^2^/day |
| Rocking | 0.008 | 0.025 | -9.53 to -.02 rads/sec/day |
| Time | 0.009 | 0.05 | -10.98 to -1.66 sec/day |
| Path Length | 0.006 | 0.007 | -6.2 to -.50 cm/day |

*Holhm- Bonferroni adjusted significance level  p value = 0.05/9=0.006 . Compare lowest p-value to adjusted p value if <0.006 go to next with new adjusted p value = 0.05/(9-p value rank+1)

Reference :

1. Aickin M, Gensler H. Adjusting for multiple testing when reporting research results: the Bonferroni vs Holm methods. Am J Public Health. 1996 May;86(5):726-8. doi: 10.2105/ajph.86.5.726.
